# Association between mobile phone use and neck pain in university students: A cross-sectional study using numeric rating scale for evaluation of neck pain

**DOI:** 10.1371/journal.pone.0217231

**Published:** 2019-05-20

**Authors:** Fadi Al-Hadidi, Isam Bsisu, Saif Aldeen AlRyalat, Belal Al-Zu’bi, Rasha Bsisu, Mohammad Hamdan, Tareq Kanaan, Mohamad Yasin, Omar Samarah

**Affiliations:** 1 Department of Special Surgery, School of Medicine, University of Jordan, Amman, Jordan; 2 School of Medicine, University of Jordan, Amman, Jordan; 3 Department of Clinical Pharmacy, School of Pharmacy, University of Jordan, Amman, Jordan; Medical University Graz, AUSTRIA

## Abstract

**Objective:**

Mobile phones are reliable devices for communication and entertainment. However, their utilization for prolonged periods in flexed neck position is linked to neck and shoulders pain. The main purpose of this study is to investigate the association between neck pain and the duration of device use, taking into consideration gender, age, and the most frequent position in which students use their devices.

**Subjects and methods:**

Based on a self-administered online questionnaire, we filled 500 questionnaires between February 15th, 2017 and March 18th, 2017. The study sample included healthy students from health care faculties regardless of their age, gender, or handedness.

**Results:**

Analysis of the predictors for pain severity showed that age (p = 0.04) and duration of use (p = 0.001) were significantly associated with the severity of neck pain, while only the duration of use was significantly associated with pain duration (p = 0.036). Subjects were divided into two groups according to the pain score, 75.8% had pain severity equal or less than 4/10 and 24.2% had pain severity more than 4/10. Of those with pain severity >4, 5.8% of students sought medical help at the emergency department and 12.4% visited clinics, compared to only 0.3% seeking medical advice at an emergency department and 4.2% visiting clinics in the group with pain severity of ≤4 (p<0.001). Regarding the use of analgesia in the two groups, 44.6% of subjects with pain severity of >4 used analgesia, compared to only 12.1% in subjects with pain severity of ≤4 (p< 0.001).

**Conclusion:**

This study demonstrates a significant positive correlation between the duration of mobile phone use and the duration and severity of neck pain. Furthermore, the increased severity of neck pain places a huge burden on the healthcare system.

## Introduction

Mobile phones are considered to be the most popular portable electronic device nowadays. Recent estimates showed that at least 77% of the world’s population has their own mobile phone [1]. The main reason mobile phone use is emerging and becoming more and more popular worldwide is that it is a reliable device for communication and entertainment [2].

In 2012, a study carried out on university students in the United States of America showed that text messaging, also known as short message service (SMS), is the most frequently used communication method [3]. Many studies have been conducted to study the correlation between using mobile phones for texting and both, neck and shoulder pain [4]. Moreover, even with other activities, prolonged neck flexion is linked to neck, shoulder, and upper extremity pain [5]. This can be explained by the static muscular load occurring with prolonged neck flexion along with the lack of support to the arms and the repetitive movement of the fingers, especially when using one hand only [2, 5–8]. The number of sent text messages has strong association with the severity of neck and shoulder pain, and even though the number of letters per messages has not been covered, it is thought that it influences the results [9].

Another aspect that was taken into consideration is the position which the person takes during mobile phone utilization. It is agreed that the best position is the sitting position with a straight neck and supporting the forearms, in addition to holding the mobile phone with both hands and to use both thumbs, and needless to say, this position should not be maintained for long periods [5, 6, 9].

Collectively, the frequency of mobile phone use, the purpose of mobile use, the degree of neck flexion while using the phone, and the body position are the main factors associated with neck and shoulder pain and its severity [5, 6, 9]. However, the effect of these factors and others on university students are poorly studied.

There are several pain scales that are used clinically to evaluate the severity of musculoskeletal pain, one of which is the Numeric Rating Scale (NRS-11), which is an eleven-point scale in which the end points are the extremes of no pain at all (score of 0) and the worst pain the patient has ever experienced (score of 10) [10]. The matter-of-fact that we need to determine a threshold value for increasing alertness to pain symptoms presents another challenge. Hartrick et al. [11] pointed that the NRS-11 pain severity score of “4” is frequently given special significance in this regard, suggesting it as a potential threshold value for pain severity in clinical practice.

The aim of this research is to study neck and shoulder pain among university students studying in the medical faculties in the University of Jordan. We will demonstrate the details of pain among students, taking into consideration the duration of device use, the purpose of its use, as well as the most frequent position in which the students use their devices. In addition we will compare between two groups of students, those with pain severity of less than or equal to four according to the Numeric Rating Scale (NRS-11), and those with pain severity of more than four, assessing how those two groups use their mobile phone, and how they manage their pain.

## Subjects and methods

### Data collection and study design

Based on a self-administered online questionnaire, we conducted a cross-sectional study on students from the University of Jordan in the Hashemite Kingdom of Jordan. The data collection took place between February 15^th^, 2017 and March 18^th^, 2017, during which 500 questionnaires were filled using Google forms as a web-based questionnaire (S1 File). Questionnaires were distributed to students by posting it on their batches’ groups on Facebook, which is an online social media and social networking service website. Students from five healthcare faculties were included: the faculty of Medicine, the faculty of Dentistry, the faculty of Pharmacy, the faculty of Nursing, and the faculty of Rehabilitation.

### Questionnaire design

First, general demographics including age, gender, and faculty were studied. Then the general conditions of mobile phone use including handedness, frequency of mobile phone use, duration, and position during use were studied. Students’ experience of neck and shoulder pain associated with mobile phone use, including the severity of the pain using the NRS-11 was evaluated; students were asked to rate their pain on a scale from 0 to 10, where zero represents “no pain at all” and 10 represents “the worst pain they have ever experienced,” using whole numbers [10]. Finally, the measures students do to relieve their pain, including changing the position, decreasing their mobile phone use, analgesia use, and seeking medical care were assessed.

### Inclusion and exclusion criteria

The study sample included students from the aforementioned five health care faculties regardless of their age, gender, or handedness. Students should have their own smart phone. On the other hand, we excluded students with any chronic medical illness, musculoskeletal diseases or anomalies, or those who had previous surgeries in the neck or shoulders. In addition, none of the faculty staff or Jordan University Hospital staff were included in the study.

### Ethical approval

The study was approved by the Institutional Review Board (IRB) of Jordan University Hospital. An informed consent was obtained in the first page of the study’s questionnaire, and it was written in Arabic, which is the official language in Jordan, it explained the aims of the study and emphasized the confidentiality of the filled information. Participants were able to withdraw from the questionnaire at any point. No identifying information were obtained through the questionnaire, and all collected data were solely used for statistical analysis.

### Statistical analysis

SPSS (version 21.0, Chicago, USA) was used in analysis of the data. Descriptive statistics were used to study the sample. One-way ANOVA and independent sample t-test were used to analyze the differences for age, duration of use, and both pain duration and severity with different factors, Tukey post-hoc analysis was used where relevant. Regression analysis was used to predict factors affecting pain severity and then to study factors affecting pain duration. A p value of 0.05 was adopted as a threshold for significance.

## Results

### Demographics

A total of 500 university students were included in this study. The mean age for study participants was 21.5 (SD = 2.6). The sample included 166 men (33.2%) and 334 women (66.8%). Most of the participating students were from the faculty of medicine (70%), followed by the faculty of pharmacy (11.6%), faculty of dentistry (9.6%), faculty of rehabilitation (5.2%), and finally the faculty of nursing (3.4%).

### Pattern of mobile phone use

Upon analyzing the duration of mobile phone use for different faculty students, a significant difference was found between them (p< 0.001), as dentistry students use mobile phones for an average of 9.8 ± 7.1 hours per day, which was significantly more than medical students (p< 0.001, mean = 5.9 ± 4.2 hours/day), nursing students (p = 0.013, mean = 5.8 ± 5.6 hours/day), and pharmacy students (p = 0.001, mean = 6.3 ± 3.5 hours/day). A significant difference between genders was also found (p = 0.005), as females tend to use mobile phones for a mean duration of 6.9 ± 4.9 hours, while males use them for a mean duration of 5.6 ± 3.9 hours. The pattern and position of mobile phone use was also analyzed between both genders, the results are summarized in **Table 1**.

**Table 1:** The difference in mobile phone use characteristics by gender.

Characteristic			Gender		Total
			Male	Female	
Position	Lying	Count	39	65	104
		% within position	37.5%	62.5%	100.0%
	Sitting	Count	114	245	359
		% within position	31.8%	68.2%	100.0%
	Standing	Count	6	20	26
		% within position	23.1%	76.9%	100.0%
	Walking	Count	7	4	11
		% within position	63.6%	36.4%	100.0%
Handedness	Right Handed	Count	146	309	455
		% within handedness	32.1%	67.9%	100.0%
	Left Handed	Count	20	25	45
		% within handedness	44.4%	55.6%	100.0%
Uni-handed or bi-handed	One Hand	Count	54	69	123
		% within uni-handed or bi-handed	43.9%	56.1%	100.0%
	Two Hands	Count	33	51	84
		% within uni-handed or bi-handed	39.3%	60.7%	100.0%

Furthermore, the duration of mobile phone use specifically for studying was analyzed with different factors. It was found that females use mobile phone for studying significantly more than males (p = 0.003), with a mean duration of 2.5 ± 2.3 hours for female students, compared to 1.7 ± 1.2 hours for male students. A significant positive correlation was also found between the duration of use for studying and the duration of pain (p< 0.001, r = 0.212). No significant differences were found between the duration of mobile use for studying and neither the age, faculty, position of use, nor the severity of pain.

### Neck pain characteristics and associated factors

**Fig 1:**
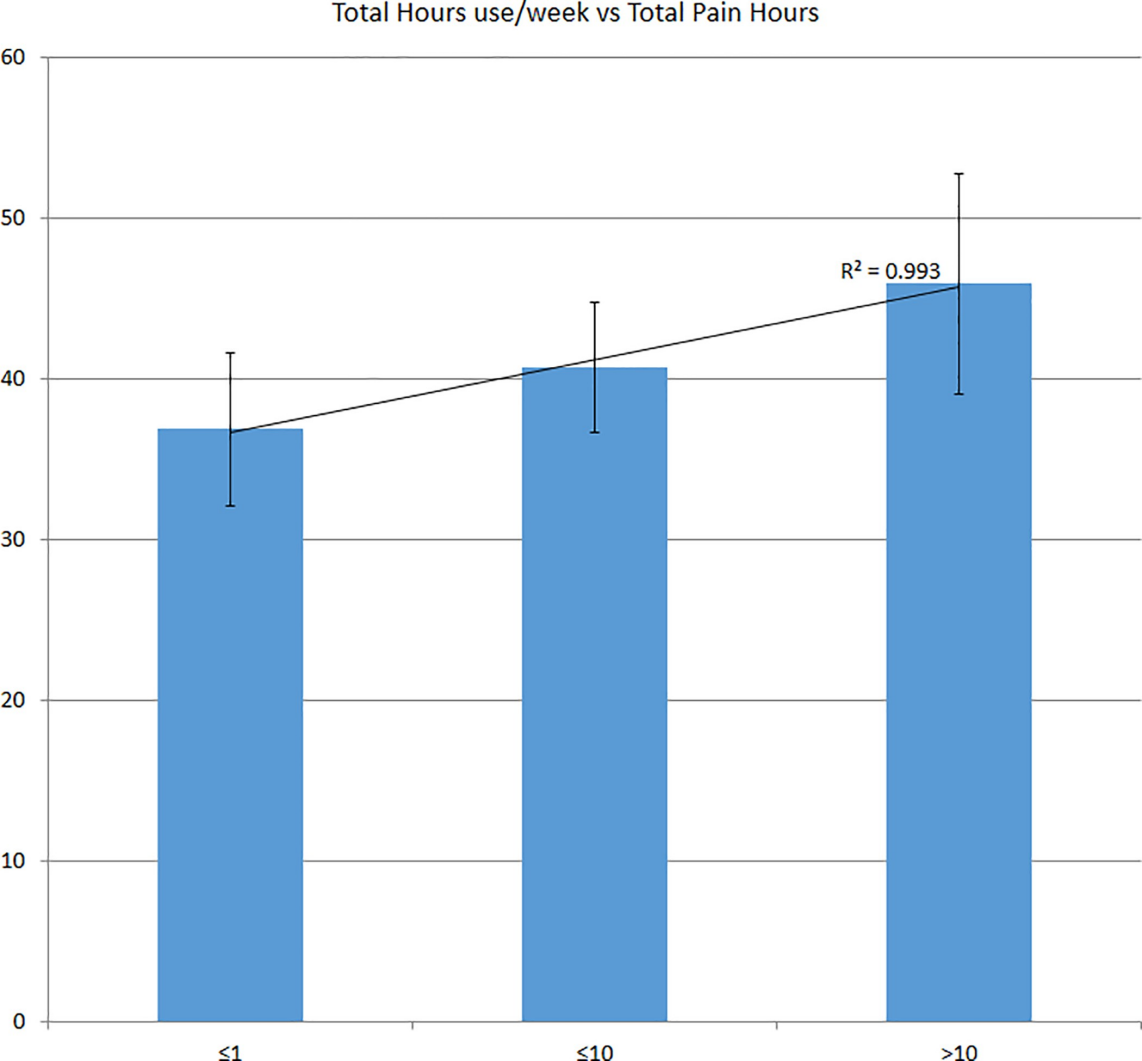
The total hours of using mobile phone and its relation to pain.

**Fig 2:**
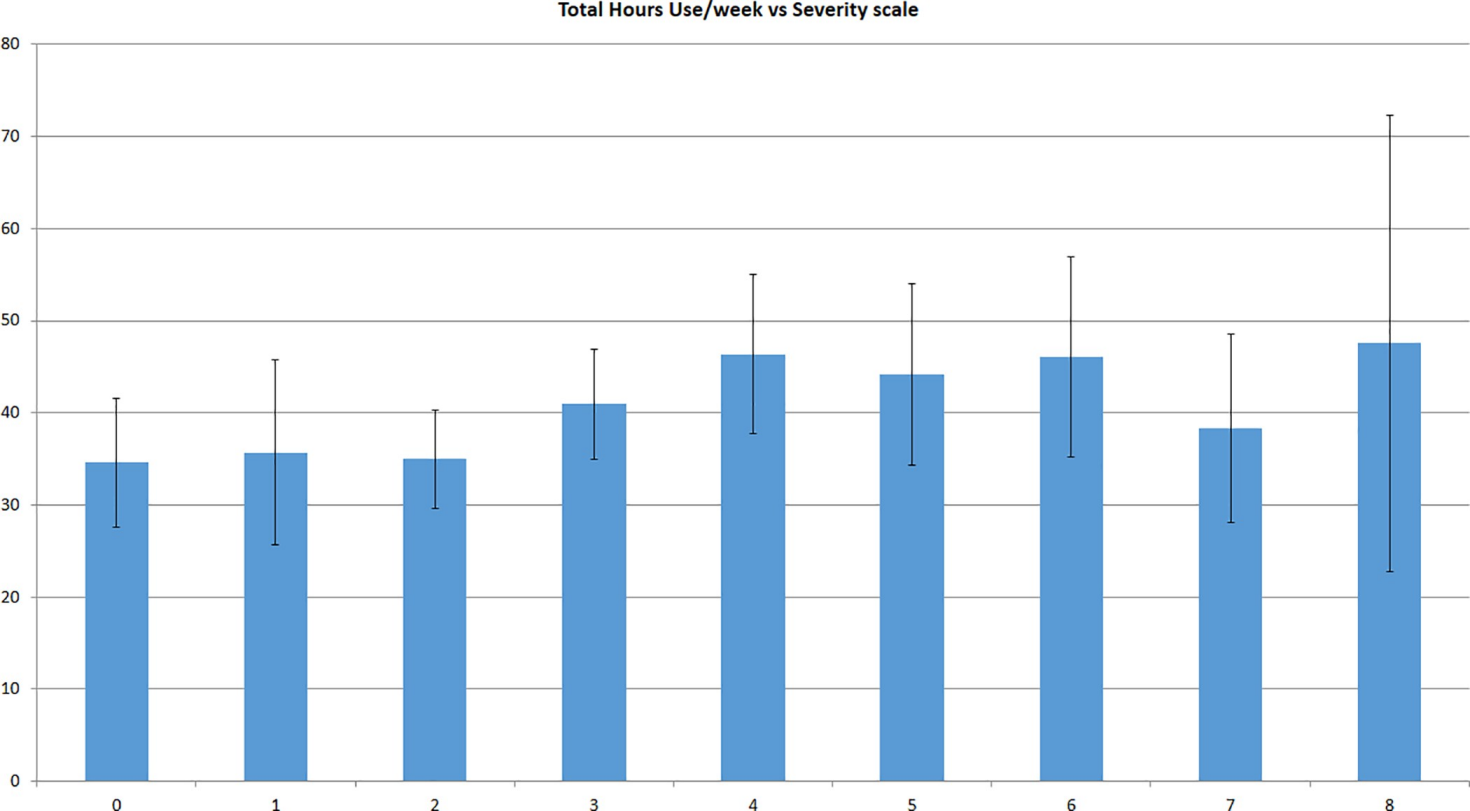
The total duration of mobile phone use per week and its relation with pain severity.

Upon analyzing predictors for pain severity, it was found that only age (p = 0.04) and duration of use (p = 0.001) were significantly associated with severity of neck pain with Pearson correlation of 0.06 for age and 0.14 for duration of use (**Fig 1**). Regarding the duration of pain, it was found that it was only significantly associated with the duration of mobile phone use (p = 0.036), with Pearson correlation of 0.1 (**Fig 2**).

Upon investigating what students generally do to decrease their pain and its association with different pain severities, a significant difference between pain severity and seeking medical care was found (p< 0.001); students with mean pain severity of 2.6 ± 2.1 do not seek medical care, while students with mean pain severity of 4.5 ± 2 visit clinics, and those with mean pain severity of 6 ± 2.6 go to emergency department. A significant difference (p< 0.001) was also found between pain severity and the use of analgesia, as students with a mean pain severity of 2.6 ± 2.1 do not use analgesia compared with students with a mean pain severity of 4.7 ± 1.9 who use analgesia. In addition, a significant difference was also found between pain severity and changing position (p< 0.001), as students tend to change their position during mobile use when they have a mean pain severity of 4.1 ± 1.5 compared to a mean pain severity of 2.6 ± 1.9 for students who do not change their position. Unfortunately, no significant relationship was found between pain severity and decreasing the use of mobile phones among our university students.

### The burden of neck pain severity on healthcare system

Of the 500 university students, 379 (75.8%) had pain severity equal or less than 4/10 and 121 (24.2%) had pain severity more than 4/10. Neither gender differences nor the field of study were significantly different between the two groups, as shown in **Table 2** and **Table 3** respectively.

**Table 2:** Comparison between genders in pain severity.

			Gender		Total
			Male	Female	
Pain severity	≤4	Count	133_a_	246_a_	379
		% within pain severity	35.1%	64.9%	100.0%
	>4	Count	33_a_	88_a_	121
		% within pain severity	27.3%	72.7%	100.0%

Each subscript letter denotes a subset of gender categories whose column proportions do not differ significantly from each other at the 0.05 level (p = 0.068).

**Table 3:** Comparison between faculties in pain severity.

			Faculty					Total
			Medicine	Dentistry	Nursing	Pharmacy	Rehabilitation	
Pain severity	<4	Count	271_a_	31_a_	14_a_	42_a_	21_a_	379
		%	71.5%	8.2%	3.7%	11.1%	5.5%	100.0%
	>4	Count	80_a_	17_a_	3_a_	16_a_	5_a_	121
		%	66.1%	14.0%	2.5%	13.2%	4.1%	100.0%

Each subscript letter denotes a subset of faculty categories whose column proportions do not differ significantly from each other at the 0.05 level (p = 0.31).

Seeking medical help was significantly different between the two groups (p< 0.001). 5.8% of students with pain severity >4 requested medical care at the emergency department and 12.4% visited outpatient clinics, compared to only 0.3% visiting emergency department and 4.2% visiting outpatient clinics in students with pain severity ≤4. In addition, the use of analgesia was significantly different between the two groups (p< 0.001), since 44.6% of patients with pain severity >4 reported using analgesia, compared to only 12.1% for patients with pain severity ≤4.

The severity of pain was a significant factor in encouraging and provoking a change in the most frequent position at which the mobile phones are used (p< 0.001), as 64% of students with pain severity >4 change their position compared to 50.4% of students with severity ≤4. Analyzing the level of awareness among students with neck and shoulders pain showed that 68.1% of them thought that their pain might be related to their use of mobile phones, while 31.9% did not believe that the pattern of their mobile phone use might be linked to their neck and shoulders pain.

## Discussion

Unsurprisingly, this study revealed that all of the student sample own smartphones. Based on their age group, these students adopt a static and flexed spinal posture while texting on mobile phones [12], which is the most common posture that contributes to neck pain [13]. To the best of our knowledge, this is the first study to use the NRS-11 pain severity score of 4 as a threshold value for increasing alertness to neck pain. In addition, it is the first study to investigate the relationship between mobile phone use and neck pain among Jordanian young adults.

A significant difference was found between gender (p = 0.005), as females tend to use mobile phones more than males (mean = 6.9±4.9 hours for females, and mean = 5.6±3.9 hours for males). It was also found that females use mobile phone for studying significantly more than males, with a mean duration of 2.5 ±2.3 hours for female students, compared to 1.7 ±1.2 hours for males. In literature, neck and shoulders pain was more frequently reported amongst females. This can be due to the general trend of females experiencing more musculoskeletal pain and more chronic pain conditions than males, which can be attributed to a lower pain threshold in women in comparison with men [14], innate differences in somatic and visceral perception [15], lower physical activity levels of female students compared to male ones in our society, as well as the tendency of females to have more mental and psychological stress than males [16], which can lower the threshold of reporting pain symptoms, or even activate the central nervous system (CNS) to varying degrees leading to the activation of muscle spindles, which can lead to painful tensional syndromes [17, 18].

The duration of mobile phone use plays a crucial role in determining the duration of neck and shoulders pain. In this study, a noteworthy positive correlation was found between duration of mobile phone use for studying and the duration of pain. In addition, the duration of mobile phones use was significantly and positively associated with the severity of neck pain. Remarkably, a meta-analysis by Xie et al [19] involving nine studies that assessed time as a risk factor found that there is no conclusive evidence on total number of hours of smartphone use and its relationship with musculoskeletal symptoms, even though this meta-analysis included six studies which revealed a significant relationship between the durations of smart phone use and musculoskeletal complaints such as the shoulders, neck and low back pain, while three studies found no association [19].

Furthermore, literature showed that the prevalence of neck pain is increasing with age [20]. Upon analyzing predictors for pain severity in this study sample, it was found that age and duration of mobile phone use are the main determinants of pain severity, while only duration of use determined the duration of the pain itself.

The NRS-11 has been broadly investigated in adults, showing great evidence of reliability and validity to measure pain sensitivity [21, 22]. Even though Visual Analog Scale (VAS), in which pain is presented on a 100-mm horizontal line by a point between the extremes of no pain at all and the worst pain the patient has ever had, is a validated ratio measure of pain [23], it has more practical difficulties which may reduce compliance. A systemic review [10] showed that both pain-rating scales, VAS and NRS-11, are reliable and valid for use in clinical practice, and that NRS-11 has good sensitivity and patients who seek a sensitive pain rating scale are more likely to choose it due to the simplicity of its application. In addition, NRS-11 is commonly used to evaluate and follow-up pain severity for patients with neck pain [24]. In the current study, neck and shoulders pain showed a significant challenge to the healthcare system when the pain severity was more than 4 according to NRS-11 [11], since 5.8% of students with pain severity >4 sought emergency department medical help and 12.4% visited outpatient clinics. In comparison, only 0.3% sought medical help at the emergency department and 4.2% visited outpatient clinics of students with pain severity ≤4. In addition, 44.6% of students with pain severity >4 used analgesia compared to only 12.1% of students with pain severity ≤4.

There are several methods of treatment used by patients to relieve neck and shoulders pain, including warming up neck muscles from time to time, applying ice and/or heat, stretching exercises, rest, message, over the counter medications (OTCs) such as Acetaminophen, cervical epidural steroid injections, trigger points injections, physical therapy, opioids and other pain medications [13]. In the current student sample, students with mean pain severity of 4.7 (SD = 1.9) or more tend to use analgesia (p< 0.001). In addition, students tend to change their position during mobile use when their mean pain severity score was 4.1 ± 1.5 (p< 0.001), since 64% of students with pain severity >4 change their position in order to alleviate the pain, compared to 50.4% of students with pain severity ≤4.

## Limitations of the study

This study has several limitations; the use of other devices (e.g. laptops) or even the posture of studying from books was not considered. Moreover, the questionnaires were filled by 500 university students from a total of 5713 students, with a response rate of 8.75%, which is half the response rate expected by online questionnaires on social media without sending messages to the study population [25, 26]. In addition, the questionnaire included only university students who use social media, and was limited to their age group, limiting the ability of this study to assess the pain characteristics among different age groups.

## Conclusions

In conclusion, smartphones are increasingly becoming essential in all aspects of our lives, and more attention should be given towards increasing awareness about the importance of having healthy sitting positions and using mobile phones for restricted durations, in order to control the increasing prevalence of neck and shoulder pain in our societies.

## Supporting information

S1 Table The study data. (XLSX)

S1 File The research questionnaire in English language. (DOCX)
