# Supplementary material for: Association between mobile phone use and neck pain in university students: A cross-sectional study using numeric rating scale for evaluation of neck pain
Source: PLoS One. 2019 May 20;14(5):e0217231. doi: 10.1371/journal.pone.0217231 (PMC6527223; doi:10.1371/journal.pone.0217231)
Supplement: S1 File — (DOCX) [file pone.0217231.s002.docx]

Smart Phone Use and its Relationship with Neck pain

Q1) What Is Your Faculty?
[ ]School of Medicine
[ ] School of Dentistry
[ ] School of Pharmacy
[ ] School of Nursing
[ ]Faculty of Rehabilitation

Q2) What Is Your Gender?

[ ] Male

[ ] Female

Q3) What Is Your Age: ________

Q4) Handedness:

"Which hand do you use for writing?"

[ ] Right Handed

[ ] Left Handed

Q5) What is the average frequency for your use of your mobile device (in days per week)? ________

Q6) What is the average time (in hours per day) that you spend using your mobile device? ___________

Q7) What is your purpose of using your mobile device?
*You Can Choose More Than One Choice

[ ] Studying

[ ] Texting

[ ] Working

[ ] Playing Games

[ ] Social Media

[ ] Watching Videos

Q8) How many Hours do you spend on your device for studying (Answer it if you have chosen studying in the previous question)? ___________

Q9) How many text messages do you send per day (Answer it if you have chosen texting in the previous question)? __________

Q10) Do you hold your mobile in one hand or both hands when you use it

[ ] One hand

[ ] Two hands

Q11) What is your most frequent position when you use your mobile device

[ ] Sitting position

[ ] Standing position

[ ] Walking position

[ ] Supine position (Lying down)

Q12) Did you experience neck or shoulder pain before?

[ ] Yes

[ ] No

Q13) What is the pain site?

*You can choose more than one choice

[ ] Neck.

[ ] Right Shoulder.

[ ] Left Shoulder.

[ ] I do not have pain.

Q14) What is the pain frequency (in days per week)

| 1 | 2 | 3 | 4 | 5 | 6 | 7 | I do not have pain |
| --- | --- | --- | --- | --- | --- | --- | --- |

Q15) What is the pain duration (in hours)? ________

If you do not have pain, please put (0).

Q16) What is the most common timing of the pain

[ ] In the morning.

[ ] At night.

[ ] In the Afternoon.

[ ] Throughout the day.

[ ] I Do not have pain.

Q17) What is the pain's severity on scale of 10? Knowing that 10 means the strongest pain you have ever experienced ________

Q18) Have you ever used analgesia to decrease this pain?

[ ] Yes.

[ ] No.

Q19) If Yes, What are the types of this Analgesic agents used?

[ ] Nonsteroidal Anti-Inflammatory Drugs (ibuprofen, panadol naproxen, and Aspirin...etc).

[ ] Corticosteroids.

[ ] Neurological Analgesia (gabapentin, amitriptyline..etc).

[ ] Opioids.

[ ] Anesthetic Nerve Blockade.

[ ] Alternatives to Analgesics: Heat.

[ ] Alternatives to Analgesics: Ice.

[ ] Alternatives to Analgesics: Massage.

[ ] Alternatives to Analgesics: Rest.

[ ] Alternatives to Analgesics: Relaxation techniques.

[ ] I Did Not Use Any Agent.

[ ] Others ( Please Specify):___________

Q20) What is the frequency of the use of analgesics agents? (In Days per week)

| 1 | 2 | 3 | 4 | 5 | 6 | 7 | I do not use analgesics |
| --- | --- | --- | --- | --- | --- | --- | --- |

Q21) Have you ever seek medical care due to this pain?

[ ] Yes, I've visited the Clinic.

[ ] Yes, I've visited the Emergency Department.

[ ] No.

Q22) Did You Decrease the use of your mobile Device after experiencing this pain

[ ] Yes.

[ ] No.

[ ] I do not have pain.

Q23) Did You Change your most frequent position while using your mobile Device after experiencing this pain

[ ] Yes.

[ ] No.

[ ] I do not have pain.

Q24) Did The pain Severity Decrease? how much would you rate the pain's severity on scale of 10, knowing that 10 means the strongest pain you have ever experienced?_________

Q25) Do you think that this pain was related to the use of your mobile device?

[ ] Yes.

[ ] No.

[ ] I do not have pain, but i believe that this type of pain is related to mobile phones use.

[ ] I do not have pain, and I Do not believe that this type of pain is related to mobile phones use.
